# DUSP6 expression is associated with osteoporosis through the regulation of osteoclast differentiation via ERK2/Smad2 signaling

**DOI:** 10.1038/s41419-021-04110-y

**Published:** 2021-09-02

**Authors:** Boya Zhang, Putao Yuan, Guang Xu, Zhijun Chen, Zhifei Li, Huali Ye, Jiying Wang, Peihua Shi, Xuewu Sun

**Affiliations:** 1 Department of Dermatology, Sir Run Run Shaw Hospital, Zhejiang University School of Medicine, Hangzhou, China; 2 Key Laboratory of Biotherapy of Zhejiang Province, Sir Run Run Shaw Hospital, Zhejiang University School of Medicine, Hangzhou, China; 3 Department of Orthopaedic Surgery, Sir Run Run Shaw Hospital, Zhejiang University School of Medicine, Hangzhou, China; 4 Key Laboratory of Musculoskeletal System Degeneration and Regeneration Translational Research of Zhejiang Province, Hangzhou, China; 5 The Affiliated Hospital of Medical School of Ningbo University, Ningbo, China

**Keywords:** Bone, Osteoporosis

## Abstract

Osteoporosis-related fractures, such as femoral neck and vertebral fractures, are common in aged people, resulting in increased disability rate and health-care costs. Thus, it is of great importance to clarify the mechanism of osteoclast-related osteoporosis and find effective ways to avoid its complication. In this study, gene expression profile analysis and real-time polymerase chain reaction revealed that DUSP6 expression was suppressed in human and mice osteoporosis cases. In vitro experiments confirmed that DUSP6 overexpression prevented osteoclastogenesis, whereas inhibition of DUSP6 by small interference RNA or with a chemical inhibitor, (E/Z)-BCI, had the opposite effect. (E/Z)-BCl significantly accelerated the bone loss process in vivo by enhancing osteoclastogenesis. Bioinformatics analyses and in vitro experiments indicated that miR-181a was an upstream regulator of DUSP6. Moreover, miR-181a positively induced the differentiation and negatively regulated the apoptosis of osteoclasts via DUSP6. Furthermore, downstream signals by ERK2 and SMAD2 were also found to be involved in this process. Evaluation of ERK2-deficiency bone marrow-derived macrophages confirmed the role of ERK2 signaling in the DUSP6-mediated osteoclastogenesis. Additionally, immunoprecipitation assays confirmed that DUSP6 directly modified the phosphorylation status of SMAD2 and the subsequent nuclear transportation of NFATC1 to regulate osteoclast differentiation. Altogether, this study demonstrated for the first time the role of miRNA-181a/DUSP6 in the progression of osteoporosis via the ERK2 and SMAD2 signaling pathway. Hence, DUSP6 may represent a novel target for the treatment of osteoclast-related diseases in the future.

## Introduction

Osteoporosis is considered a degeneration-related disease that leads to low bone mass, micro-architectural deterioration of bone tissue, and bone fragility. Osteoporotic fracture (fragility fracture) is common in osteoporosis patients with bone deterioration or low bone mass [1]. Osteoporotic fracture could be caused with low-energy trauma, such as a minor fall or during routine activities. Hip and vertebral fractures are prototypical osteoporotic events, resulting in increased disability rates and high health-care costs [2]. A previous study indicated that approximately 2.7 million hip fractures occurred in 2010 worldwide, and if osteoporosis could be treated, 51% of these fractures could be prevented [3]. Therefore, it is essential to clarify the underlying mechanism of osteoporosis and identify novel and effective targets for osteoporosis treatment.

The current treatment options for osteoporosis either antagonize bone resorption or promote bone formation [4, 5]. However, all medications, either new (such as cathepsin K inhibitors, Src kinase inhibitors, vitamin D analogs, and chloride channel inhibitors) or already approved by the U.S. Food and Drug Administration (such as bisphosphonates, denosumab, calcitonin, strontium ranelate, hormone replacement therapy drugs, selective estrogen receptor modulators, and recombinant human thyroid paragon hormone-related drugs), have various side effects. Denosumab, as the strongest anti-resorptive to date, is widely used in osteoporosis patients. It binds to and inhibits RANK ligand, resulting in significant inhibition of bone remodeling; however, denosumab long-term treatment was reported to be associated with skin rash and infection, and atypical femoral fractures and osteonecrosis of the jaw [6]. Abaloparatide, a synthetic analog of the parathyroid hormone-related protein, reduced the nonvertebral fractures but increased the heart rate and palpitations [7]. Thus, it is necessary to elucidate the regulation of bone resorption and formation to safely and efficiently prevent and treat osteoporosis.

Dual-specificity phosphatases (DUSPs) are major signaling modulators in various physiological and pathological processes [8]. For instance, the DUSP/p38/IRa signaling pathway regulates the chamber-specific perinatal growth in the heart [9]; DUSP12, along with ASK1, acts as a positive regulator in hepatic steatosis [10]; DUSP26 and DUSP9 protect against non-alcoholic fatty liver disease [11, 12], and hypoxia-induced downregulated DUSP2 regulates colon cancer stemness [13]. DUSP6 has been extensively studied and was shown to participate in various biochemical processes as a cytoplasmic enzyme that dephosphorylates/inhibits the extracellular signal-regulated kinase (ERK)1/2 [14]. Previous studies also indicated that DUSP6-mediated SUMOylation protects cells from oxidative damage [15]. Moreover, DUSP6, along with DUSP1, suppresses malignant peripheral nerve sheath tumor (MPNST) growth via JNK signaling [16]. In T cells, DUSP6 mediates receptor-engaged glycolysis and restrains differentiation [17]. DUSP6 overexpression attenuates the development of graft-versus-host disease [18], whereas DUSP6 deficiency regulates the gut microbiome in mice [19]. However, the role of DUSP6 in osteoclast-related diseases remains unknown.

MicroRNAs (miRNAs) regulate gene expression and modulate several physiological and pathological conditions [20, 21], playing a central role in the progress of musculoskeletal diseases, including osteoporosis, osteoarthritis, and intervertebral disc degeneration [22]. MiR-181a-5p is a critical mediator in the destruction of lumbar facet joint cartilage [23], whereas miR-181a-5p antisense oligonucleotides can limit articular cartilage degeneration in vivo [23]. Previous studies indicated that miR-181a modulated Fasl expression and affected CD4+ T lymphocyte and osteoclast apoptosis to maintain bone remodeling balance [24, 25]. However, the specific function and underlying mechanism of miR-181a in the progress of osteoporosis remains unclear.

Herein, we demonstrate that DUSP6 is significantly downregulated in osteoporotic bone tissue. Overexpression of DUSP6 significantly inhibits osteoclast differentiation, whereas inhibition of DUSP6 by small interference RNA (siRNA) or the inhibitor (E/Z)-BCI has the opposite effect. MiR-181a was found to suppress DUSP6 expression and regulate the subsequent signaling pathway. In addition, DUSP6 was found to bind to SMAD2 and suppress its phosphorylation. Given the special effect of DUSP6 on osteoclastogenesis, along with the present findings, DUSP6 might be a novel target for treating osteoporosis or rheumatoid arthritis.

## Material and methods

### Mice and reagents

ERK2f/f mice on a C57BL/6J background were purchased from Jackson Laboratories (Stock No: 019112). Dulbecco’s modified Eagle’s medium and fetal bovine serum were purchased from Gibco-BRL (Gaithersburg, MD, USA). DUSP6 inhibitor (E/Z)-BCI was purchased from Medchem Express (USA), and it was dissolved in DMSO and stored in −20 °C. DMSO was used less than 0.1% to the total culture medium in the indicated experiments. Antibodies specific for DUSP6 (ab76310), Cathepsin K (ab37259), ACTIN (ab6276), C-FOS (ab222699), and GAPDH (ab8245) were purchased from Abcam (Cambridge, MA, USA). Antibodies specific for NFATC1(#8032), P-ERK (#4370), ERK (#4695), P-JNK (#4668), JNK (#9252), P-P38(#4511), P38(#8690), P-SMAD2(#18338), and SMAD2 (#5339) were purchased from Cell Signaling Technology (Danvers, MA, USA). DUSP6 (sc-377070) was purchased from Santa Cruz Biotechnology. Adenovirus-mediated overexpression of DUSP6 was generated according to the previous study [26]. RNA interference, miR-181a mimic, miR-181a inhibitor, non-specific control miR (NC), and miR-NC inhibitor were synthesized by Shanghai GenePharma (Shanghai, China). All other chemicals used were of analytical grade and indicated in the article.

### Human vertebral tissue and experimental osteoporosis in mice

Human osteoporoticl vertebral tissue (*N* = 10) was collected in patients suffering from vertebral fractures at the time of percutaneous vertebroplasty. Human normal vertebral tissue (*N* = 10) was collected in patients suffering from lumbar disc herniation at the time of percutaneous endoscopic lumbar discectomy. BMD *T*-score (−2.5 or lower) was identified as osteoporosis [27]. Collecting human materials has been approved by the institutional review board of the Ethics Committee of Sir Run Run Shaw Hospital, Zhejiang University. And the informed consents have been obtained before the study.

The animal experiments carried out in our study was according to the principles and procedures of the Guide for the Care and Use of Laboratory Animals (NIH) and the guidelines for animal treatments of Sir Run Run Shaw Hospital. And the experimental protocols have been approved by the Ethics Committee of Sir Run Run Shaw Hospital. The ovariectomy-induced osteoporosis model was established according to the previous study to explore the role of DUSP6 inhibitor (E/Z)-BCI. Briefly, 12-week-old female C57BL/J6 mice were subjected to either a sham surgery or bilateral ovariectomy and assigned randomly to three groups (*n* = 5): sham-operated, ovariectomy with DMSO (OVX + DMSO), and ovariectomy with (30 mg/kg body weight, OVX + (E/Z)-BCI). One week after operation, mice were injected intraperitoneally with (E/Z)-BCI twice a week. The other two groups were injected with 1% DMSO. All mice were sacrificed 6 weeks after ovariectomy operation. The collected bone tissue was used for histological and micro-CT (micro-computed tomography) analysis.

Ovariectomy-induced osteoporosis (OVX group and SHAM group) model was established. Adenovirus-DUSP6 and -Ctrl, at a dose of 1 × 10^8^ units, was injected into the tail vein of mice once every 2 weeks for a total of 8 weeks [28]. All mice were euthanized and the collected bone tissue was used for histological and micro-computed tomography analysis.

### Histological analysis

Left femurs and tibias from each mouse group were fixed in 4% buffered paraformaldehyde for 24 h, then submerged in 10% EDTA (w/v) with phosphate-buffered saline to decalcify for about 2 weeks, and finally embedded in paraffin. Each specimen was sectioned with a microtome at a thickness of approximately 4 μm. The sections were treated with TRAP staining (Sigma-Aldrich, St. Louis, MO, USA) for osteoclast analysis. For DUSP6 analysis, immunofluorescence experiments were performed as described previously [29]. Briefly, the sections were incubated with DUSP6 (1:100), and a secondary antibody conjugated with Alexa Fluor 568 (1:500) and DAPI (1:1000) was used for fluorescence microscopy. The fluorescence signal intensity was analyzed using ImageJ (National Institutes of Health, Bethesda, MD, USA). Bone volume/tissue volume (BV/TV), bone surface/bone volume (BS/BV), mean trabecular thickness (TbTh), and bone mineral density (BMD) of fixed tibiae were analyzed using a high-resolution micro-CT instrument (Bruker, Billerica, MA, USA), as described previously [29].

### Microarray analysis

Total RNA was extracted with TRIzol Reagent (Thermo Fisher Scientific, Waltham, MA, USA) and analyzed by Genesky Biotechnologies Inc. (Shanghai, P.R. China). Briefly, total RNA quality was verified using an Agilent 2100 Bioanalyzer (Agilent Technologies, Santa Clara, CA, USA). The mRNA were fragmented and purified using AgencourtAMPure XP-PCR Purification Beads (Beckman Coulter, Brea, CA, USA). Double size selection was performed using AgencourtSPRIselect Reagent Kit (Beckman Coulter). A Qubit and Agilent 2100 Bioanalyzer was used to check the quality of the short fragment library; RNA-sequencing was performed on an Illumina HiSeq platform (Illumina, San Diego, CA, USA) with 2 × 150 bp. The raw sequencing reads were evaluated by FastQC (V0.11.4) The KEGG and GO enrichment analysis was performed as previously described [30].

### Predicted miRNA of DUSP6

MiRNAs that bind to DUSP6 were predicted using the bioinformatics databases TargetScan (http://www.targetscan.org/) and miRDB (http://www.mirdb.org/) and the published microRNA biomarker for postmenopausal osteoporosis (GSE64433). The overlapping miRNAs were considered reliable miRNA controlling DUSP6.

### Luciferase assay

The 293T cell line was obtained from ATCC. The wild-type ormutated DUSP6 of the predicted binding site of miR-181a was generated according to the previous study [31]. Five hundred nanograms of DUSP6-WT or DUSP6R-Mut plasmid and 50 nmol of miR-181a minic or negative control were transfected using Lipofectamine 2000 (Invitrogen) according to the manufacturer’s protocol in 293T cell line. Following 48 h of transfection, the cells were lysed and subjected to analysis by the dual-luciferase reporter assay system (Promega, San Luis Obispo, CA). Renilla luciferase activity was normalized to Firefly luciferase activity in each experiment.

### Mouse bone marrow-derived macrophages (BMMs) preparation and osteoclast differentiation

Primary BMMs were collected from 6-week-old male C57BL/6 mice. For osteoclast differentiation, BMMs were treated with 30 ng/ml M-CSF and 50 ng/ml RANKL for about 6 days until it differentiated into mature osteoclasts. Culture medium was changed every other day with new M-CSF and RANKL. To analyze osteoclast maturation, TRAP staining was performed as described previously [29].

### Resorption pit assay

To test the function of osteoclasts, resorption pit assay was performed, as described previously [29]. Resorption pits in bovine bone slices were visualized under a scanning electron microscope (FEI Instruments, Hillsboro, OR, USA) and quantified using ImageJ.

### F-actin ring formation staining

RANKL-induced osteoclast differentiation was observed in each mouse group for 6 days. Cells were then fixed with 4% buffered paraformaldehyde and stained with Phalloidin-iFluor™ 680 Conjugate (AAT Bioquest, Sunnyvale, CA, USA), according to the manufacturer’s protocol. The nuclei were stained with DAPI, and the cells were observed under a fluorescence microscope.

### Quantitative real-time PCR

The cultured cells from different groups and bone tissues were lysed with TRIzol reagent (Invitrogen, Carlsbad, CA) to obtain RNA or miRNA, according to the manufacturer’s protocol. The reactions were normalized to the miRNA with U6 or the mRNA with b-actin. The primer sequences were used in this study according to a previous report unless otherwise noted [29].

Human DUSP6:

F: GAAATGGCGATCAGCAAGACG; R: CGACGACTCGTATAGCTCCTG

Mouse DUSP6:

F: ATAGATACGCTCAGACCCGTG; R: ATCAGCAGAAGCCGTTCGTT

Mouse ERK2f/f

F: AGCCAACAATCCCAAACCTG; R: GGCTGCAACCATCTCACAAT (ERK2+/+275 bp and ERK2f/f 350 bp)

Mouse TRAP:

F:CACTCCCACC-CTGAGATTTGT; R:CCCCAGAGACATGATGAAGTCA

Mouse Nfatc1

F:CCGTTGCTTCCAGAAAATAACA; R:TGTGGGATGTGAACTCGGAA

Mouse DC-STAMP

F:AAAACCCTTGGGCTGTTCTT; R:AATCATGGACGACTCCTTGG

Mouse c-Fos

F:CC-AGTCAAGAGCATCAGCAA; R:AAGTAGTG-CAGCCCGGAGTA

### Immunoprecipitation and western blotting

Proteins from each mouse group were extracted using RIPA lysis buffer (Sigma-Aldrich, St. Louis, MO, USA), according to the manufacturer’s protocol. The obtained lysates were then centrifuged at 12,000 r.p.m. for about 20 min at 4 °C. After centrifugation, the supernatants were collected for further experiments. Equal amounts of protein (20 mg of each sample) were separated by 10% sodium dodecyl-sulfate polyacrylamide gel electrophoresis and electroblotted onto polyvinylidene difluoride membranes (MilliporeSigma, Burlington, MA, USA). Then, the membranes were blocked with 5% (w/v) bovine serum albumin for about 2 h, and incubated with primary antibodies at 4 °C overnight. The membranes were washed with 0.1% Tween-20 in Tris-buffered saline and incubated with secondary antibodies for 2 h at room temperature. Afterward, the immunoreactive bands were detected using enhanced chemiluminescent detection reagent (MilliporeSigma) on the XRS chemiluminescence detection system (Bio-Rad, Hercules, CA, USA). The gray levels of bands were quantified using ImageJ (National Institutes of Health, Bethesda, MD, USA). To explore the association between phospho-SMAD2 and DUSP6, immunoprecipitation experiments were performed, as described previously [26]. RAW264.7 cells were seeded in 6-cm dishes and stimulated with 50 ng/ml RANKL for 30 min. The lysates were collected and centrifuged as described above. DUSP6 antibody was added to the supernatant before incubating at 4 °C overnight. The sample was then incubated with protein A/G agarose beads (Thermo Fisher Scientific) for about 4 h at 4 °C with gentle rotation. Proteins were then analyzed by western blotting.

### Cell transfection

BMMs were transfected with adenovirus, RNAi, or miRNAs, as described previously [26]. BMMs were transfected with the noted SiRNA using Lipofectamine 3000 (Invitrogen, Waltham, MA, USA). Briefly, the day before transfection, BMMs were seeded in six-well plates at the density of about 20,000 cells/well and transfected with about 20 nM siRNA. Six hours later, the medium was replaced with normal α-MEM containing 10% fetal bovine serum and 30 ng/ml M-CSF. After 48 h, the efficiency of transfection was verified by fluorescence microscopy. Western blot analysis was performed to further determine the efficiency of the transfection. The selected sequence of DUSP6 siRNA was 5-UGGCUUACCUUAUGCAGAA-3. Transfected BMMs were then treated with M-CSF and RANKL with the indicated treatment for further analysis.

### Statistical analysis

Data are presented as mean ± standard deviation. Statistical differences were identified by two-sample *t*-test or analysis of variance in conjunction with Student’s *t*-test. In addition, *p* < 0.05 was considered to indicate statistical significance.

## Results

### Gene microarray identified the altered gene expression of DUSP6 in osteoporotic bone tissues

**Fig. 1:**
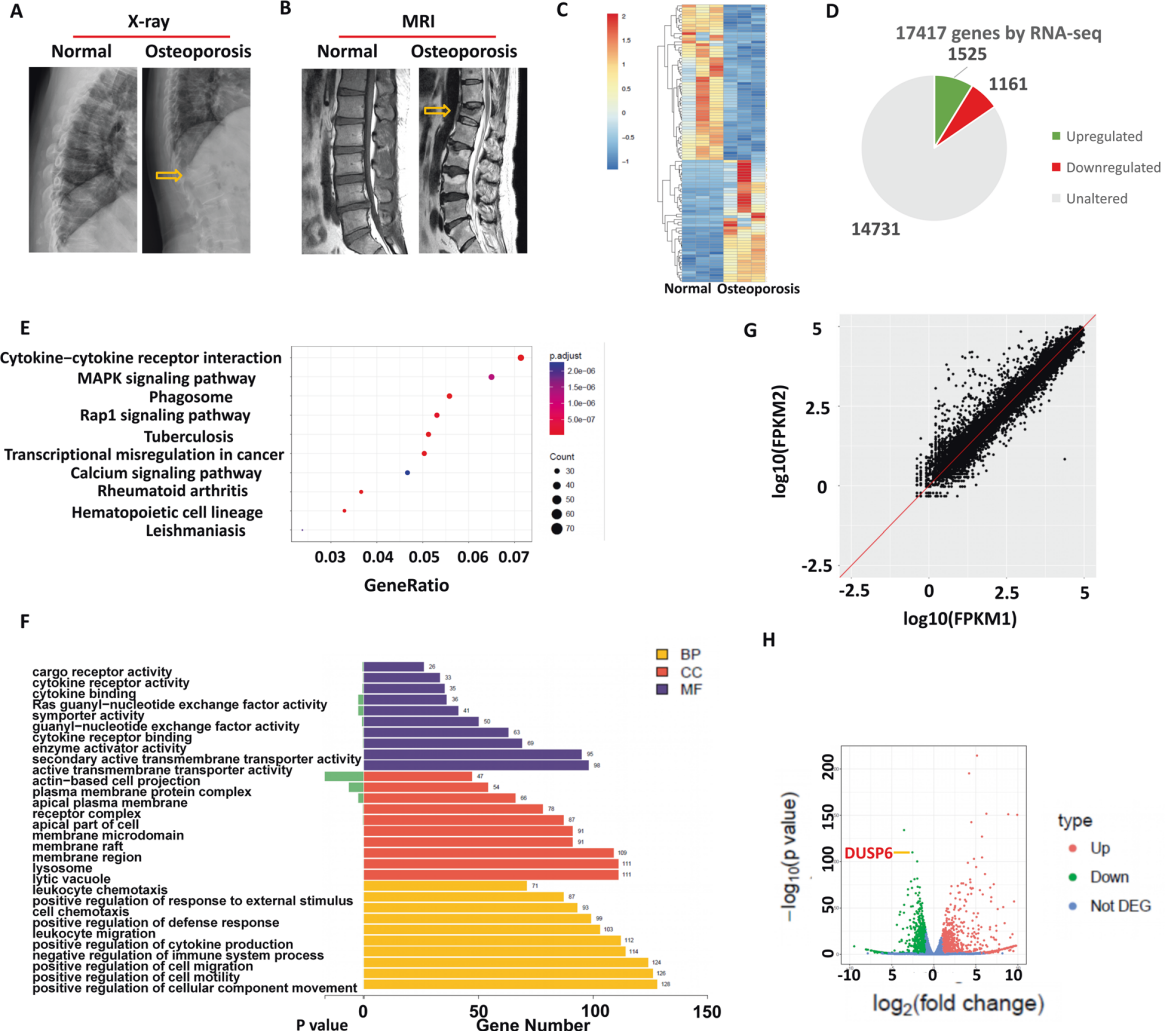
Gene microarray identified the altered gene expression of DUSP6 in osteoporotic bone tissues. Normal and osteoporosis patients were arranged for X-ray (**A**) and MRI (**B**). Bone tissues form vertebral body were collected from the two groups (*n* = 3). Gene microarray analysis was carried out to explore the differential gene expression and shown as heat map (**C**). The number of upregulated and downregulated genes were shown (**D**). KEGG (Kyoto Encyclopedia of Genes and Genomes) analysis were shown to identify the significant signaling pathway (**E**). GO enrichment analysis were carried out. CC cellular component, MF molecular function, BP biological process (**F**). The differential gene expression scatter plot in the two group was shown as log FPKM (Fragments Per Kilobase Million) (**G**). **H** The volcano map of differential genes was shown. The dot of Dsup6 was shown (red arrow).

We first collected the bone tissues from individuals with normal bone marrow density (BMD) and osteoporotic patients with vertebral fracture. X-ray and magnetic resonance imaging were performed to evaluate the vertebral body (Fig. 1A, B). Gene microarray analysis was performed and the differentially expressed genes (DEGs) between the two groups were identified, among which 1525 genes were upregulated and 1161 were downregulated (Fig. 1C, D). Kyoto Encyclopedia of Genes and Genomes (KEGG) analysis revealed that the cytokine–cytokine receptor interaction and MAPK signaling pathway were highly altered (Fig. 1E). Gene ontology (GO) enrichment analysis identified the top 10 DEGs covering three aspects of biology: cellular component, molecular function, and biological process (Fig. 1F). The DEG scatter plot is shown in Fig. 1G. A volcano map of the identified DEGs showed that XPR1, SLC4A2, and GNPTAB were the most enhanced genes, whereas GRAMD1B, ITGA6, and DUSP6 the most suppressed genes (Fig. 1H). XPR1, SLC4A2, and GNPTAB were previously reported to be related to osteoporosis, but GRAMD1B, ITGA6, and DUSP6 have never been studied with respect to any musculoskeletal-related diseases. Based on polymerase chain reaction (PCR) results, DUSP6 was suppressed the most and was thus considered the candidate gene for further investigations (Supplementary Fig. S1A).

### DUSP6 is downregulated in human and experimental osteoporosis samples

**Fig. 2:**
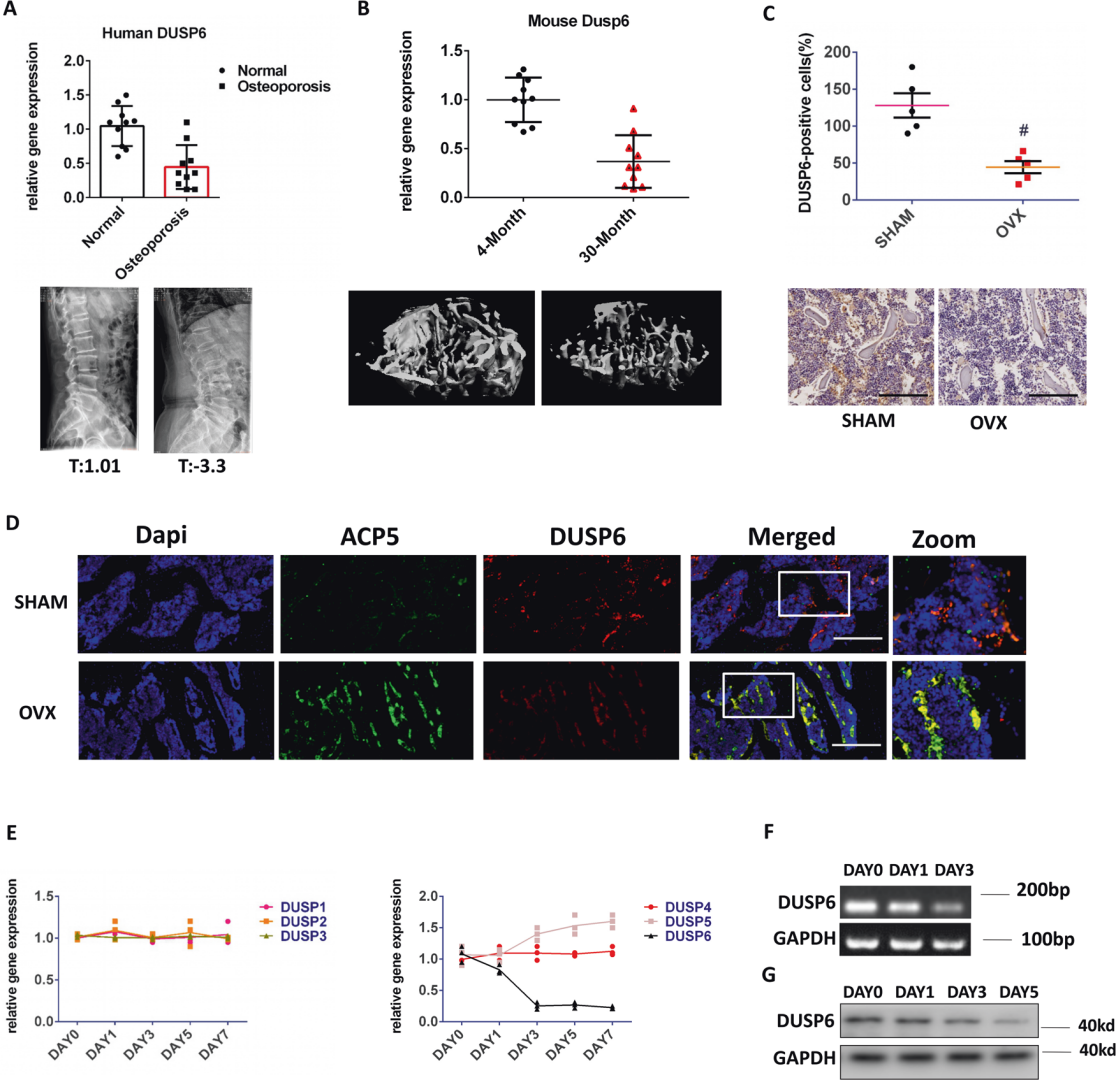
DUSP6 was downregulated in human and experimental osteoporosis samples. **A** Human normal and osteoporosis samples were collected (*n* = 10). The bone density of vertebral body was analyzed. The *T* value of human normal samples were ranged from (0.2 to 2.1, average1.01). The *T* value of human osteoporosis samples were ranged from (−2.6 to −5.2, average −3.3). We isolated the RNA from the vertebral body for PCR analysis to explore the expression of DUSP6. **B** We fed the mice until 30 months old. The tibia was collected for uCT to explore the bone loss. And the expression of DUSP6 was analyzed in the tibia from the 30-month-old and 4-month-old mice (*n* = 10). **C** Experimental osteoporosis murine model was established (*n* = 5). The tibia was collected from the two groups. Immunohistochemistry assay was carried out to explore the expression of DUSP6. Original scale bars: 500 μm. **D** The tibia was collected from the SHAM and OVX groups. Immunofluorescence assay was carried out to detect the expression and location of ACP5 (green) and DUSP6 (red). The ACP5-positive cells were regarded as an osteoclast marker. Original scale bars: 500 μm. **E** RANKL-induced osteoclast differentiation was carried out. We explored the expression of DUSP1 to DUSP6 during this process. Original scale bars: 200 μm. **F** Nucleic acid gel electrophoresis and **G** western blot analysis was employed to confirm the mRNA and protein level during the osteoclast differentiation (*n* = 3). Data in all bar graphs are expressed as mean ± SD. *P < 0.05, ^#^*P* < 0.01.

Average T values of bone density in normal and osteoporosis human samples were 1.01 and 3.3, respectively. Importantly, DUSP6 was found to be significantly downregulated in osteoporotic bone tissues (Fig. 2A). The age-related osteoporosis model was established. Bone loss was confirmed by ultrasound computed tomography (uCT). The inhibited expression of DUSP6 was observed in 30-month-old mice (Fig. 2B). We then established an ovariectomy (OVX)-induced osteoporosis murine model, and DUSP6 was suppressed in the osteoporosis group, as confirmed by immunohistochemistry (Fig. 2C). Immunofluorescence assay confirmed the co-staining of ACP5 and DUSP6 in the bone tissues of control (SHAM) and ovariectomy (OVX) groups (Fig. 2D). We then explored the expression of DUSP family members during osteoclast differentiation by real-time (RT)-PCR. DUSP1, DUSP2, DUSP3, and DUSP4 did not show any changes and DUSP5 was slightly upregulated, whereas DUSP6 was significantly downregulated (Fig. 2E). Nucleic acid gel electrophoresis and western blotting confirmed the inhibition of DUSP6 during osteoclastogenesis (Fig. 2F, G).

### Inhibition of DUSP6 in primary BMMs accelerates osteoclast differentiation in vitro

**Fig. 3:**
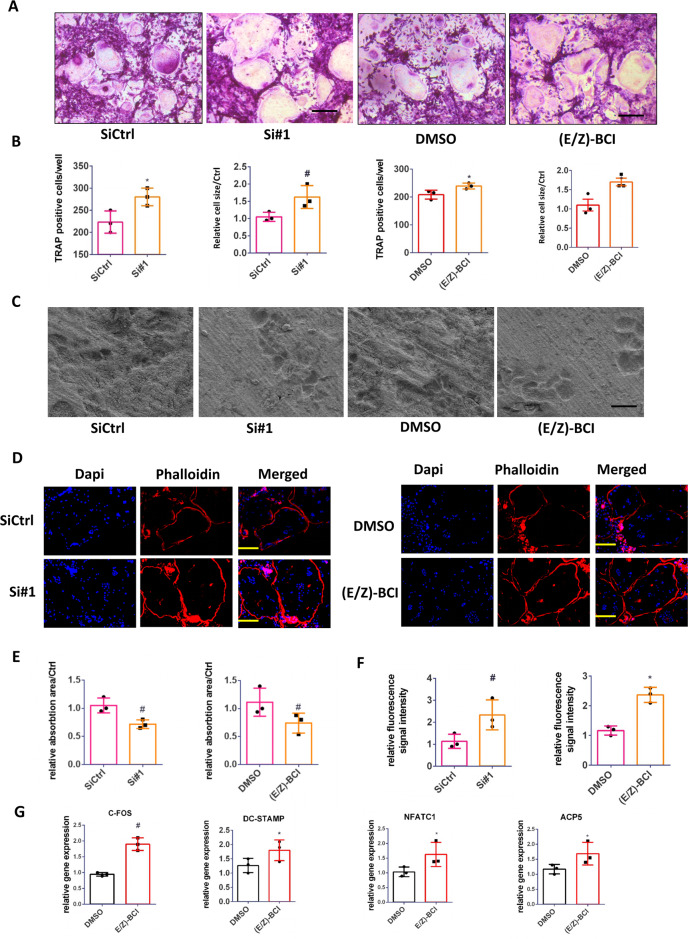
Inhibition of DUSP6 in BMMs accelerated the osteoclast differentiation in vitro. **A** RANKL-induced osteoclast differentiation was established. We silenced DUSP6 using small interfering RNA (RNAi, #1) or DUSP6 inhibitor (E/Z)-BCI (1 μM). TRAP staining was carried out to explore the differentiation of osteoclast. Original scale bars: 200 μm. **B** TRAP-positive cells number in per well and relative osteoclast cells were quantitative analyzed. **C** Bone resorption analysis was used to explore the effect of RNAi or (E/Z)-BCI on the function of osteoclasts. Original scale bars: 200 μm. **D** F-actin ring formation was carried out between the siControl(siCtrl), siDUSP6(si#1), DMSO and (E/Z)-BCI groups. The actin rings were detected using phalloidin with fluorescence microscopy. The fluorescence intensity was calculated using Imagej. Original scale bars: 200 μm. Quantitative analysis of bone resorption analysis (**E**) and F-actin ring formation (**F**). **G** The osteoclast-related gene expression of NFATc1, C-FOS, ACP5, and DC-STAMP was analyzed (day 5) using real-time PCR in the control and (E/Z)-BCI-treated group. Data in all bar graphs are expressed as mean ± SD (*n* = 3). *P < 0.05, ^#^*P* < 0.01.

Western blot analysis confirmed that DUSP6 expression was suppressed the most in the siRNA 1# group (Supplementary Fig. S1B). RANKL-induced osteoclast differentiation was demonstrated, and TRAP staining confirmed that downmodulation of DUSP6 by small RNA interference (RNAi) or chemical inhibition with (E/Z)-BCI-enhanced osteoclastogenesis. These results were confirmed by quantitative analysis (Fig. 3A, B). Bone resorption assay indicated that inhibition of DUSP6 suppressed the function of osteoclasts (Fig. 3C). F-actin ring formation performed to evaluate osteoclast maturation showed more intense signals in siDUSP6- or (E/Z)-BCI-treated groups (Fig. 3D), which was confirmed by quantitative analysis (Fig. 3E, F). We also analyzed the osteoclast-related gene expression and found that NFATC1, C-fos, ACP5, and DC-STAMP were upregulated in (E/Z)-BCI-treated group (Fig. 3G).

### DUSP6 inhibitor accelerates bone loss in vivo

**Fig. 4:**
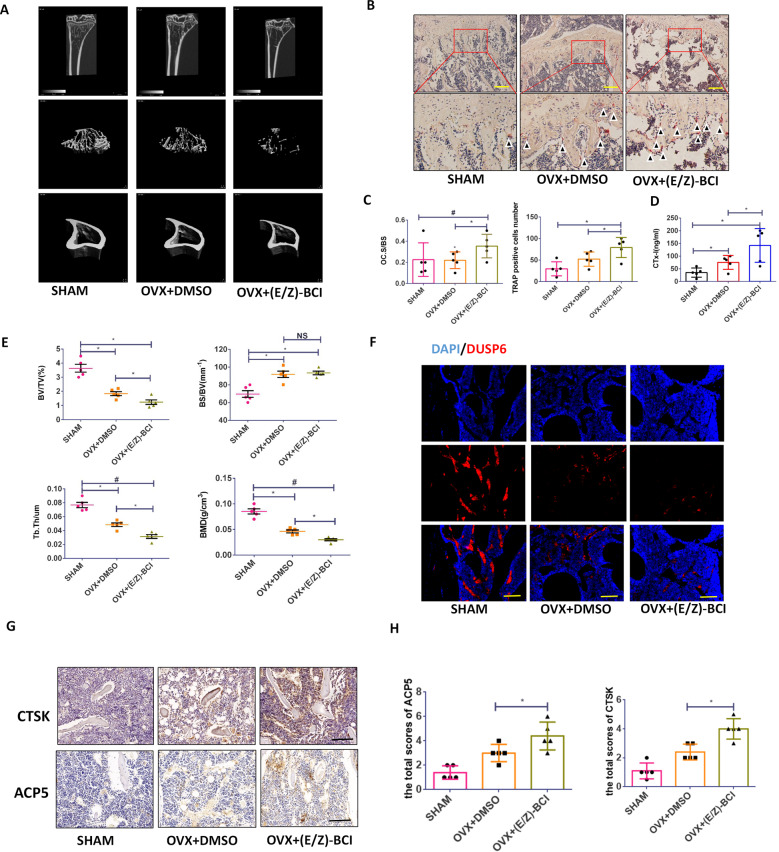
DUSP6 inhibitor accelerated the bone loss in vivo. Experimental osteoporosis model was established. Mice were treated with (E/Z)-BCI for analysis the function of DUSP6 in vivo. **A** uCT was analyzed to confirm the bone loss. **B** The tibia was collected from the indicated groups and TRAP staining was analyzed to detect the osteoclasts. Original scale bars: 500 μm. **C** Quantitative analysis was used to determine the average TRAP-positive cell numbers from five different versions and the TRAP-positive cell bone surface/bone surface. **D** Serum cross-linked C-telopeptide 1 levels were determined by ELISA (mouse CTx-I ELISA kit; Cusabio, Wuhan, China). **E** The bone volume/tissue volume (BV/TV), trabecular thickness (Tb.Th), trabecular number (Tb.N), and trabecular separation (Tb.Sp) were measured to evaluate the microstructure. **F** The tibia was collected from the indicated groups. Immunofluorescence assay was carried out to explore the expression of DUSP6 in the indicated groups. Original scale bars: 500 μm.The tibia was collected from the indicated groups. Immunohistochemistry and quantitative analysis were carried out to explore the expression of ACP5 and CTSK, which were identified as osteoclast markers (**G**, **H**). Original scale bars: 500 μm. Data in all bar graphs are expressed as mean ± SD (*n* = 5). *P < 0.05, ^#^*P* < 0.01.

To explore the function of DUSP6 in vivo, we established an experimental osteoporosis murine model. Inhibition of DUSP6 with (E/Z)-BCI significantly accelerated bone loss in mice, as confirmed by uCT (Fig. 4A). TRAP staining and quantitative analysis further showed that the number of TRAP-positive cells was higher in the (E/Z)-BCI-treated group than in the untreated osteoporosis group (Fig. 4B, C). (E/Z)-BCI treatment enhanced CTX-I expression (Fig. 4D). Quantitative analysis showed that (E/Z)-BCI treatment enhanced the bone surface density (BS/BV) but suppressed the bone volume fraction (BV/TV), trabecular thickness (Tb.Th), and BMD (Fig. 4E). Moreover, the fluorescence intensity of DUSP6 in the (E/Z)-BCI-treated group was lower than that in the untreated osteoporosis group (Fig. 4F). Immunohistochemistry confirmed the enhanced expression of ACP5 and CTSK in (E/Z)-BCI-treated group (Fig. 4G, H).

### MiR-181a regulates DUSP6 expression in osteoporotic bone tissues

**Fig. 5:**
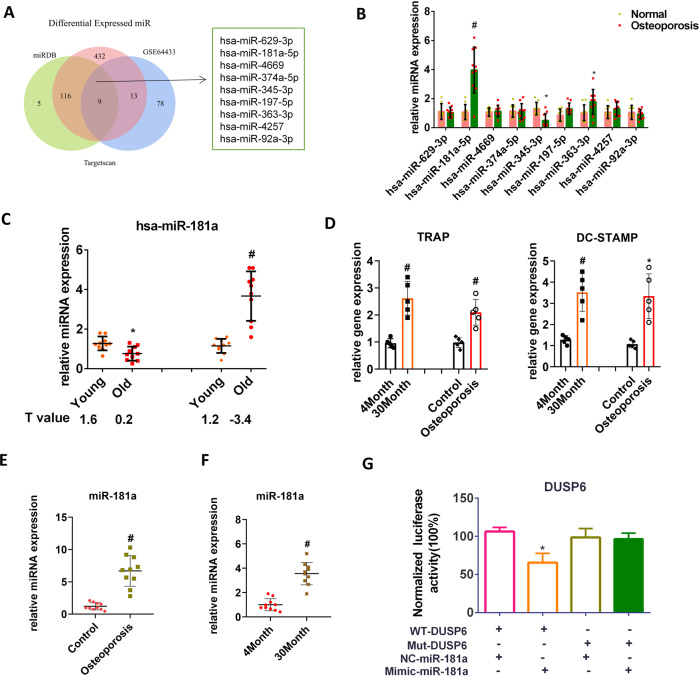
MiR-181a regulated the DUSP6 gene expression in osteoporotic bone tissues. **A** Bioinformatics approach using existed databases was used to find the regulator of DUSP6. Two databases (TargetScan and miRDB) were used to explore the potential miRNAs regulating the DUSP6. And the GSE64433 form the NCBI was used to shown the differential miRNAs between osteoporosis and normal group. The overlapping miRNAs were identified as potential regulator of DUSP6 during the osteoporosis condition. **B** Real-time PCR was carried out to confirm the nine miRNAs in human osteoporotic and normal bone tissues. *n* = 10. **C** Human samples were collected and classified as young (range from 22 to 58 years old) and old (range from 62 to 87 years old) person. And they were then divided as normal and osteoporosis groups. The average *T* value was shown in the indicated group. And the hsa-miR-181a (has-miR-181a-5p) expression was evaluated using real-time PCR. Ovariectomy-induced osteoporosis model (**D**) and age-related osteoporosis model (**E**) was established to explore the expression of miR-181a (mmu-miR-181a-5p). **F** mRNA was isolated in the tibia in mice from the indicated group. The expression of osteoclast-related gene was analyzed using real-time PCR. **G** Normal and mutated luciferase reporter containing putative miR-181a target sites of DUSP6 was established. The luciferase reporter vectors were cotransfected into HEK293 cells with miR‐181a mimics/inhibitor. The luciferase activity was quantified. Data in all bar graphs are expressed as mean ± SD. *P < 0.05, ^#^*P* < 0.01.

To clarify the regulatory mechanism of DUSP6, a bioinformatics approach was applied using TargetScan, miRDB, and published miRNA biomarkers for postmenopausal osteoporosis (GSE64433 dataset). Nine overlapping miRNAs were identified (Fig. 5A). PCR was performed in human osteoporotic bone tissues to confirm the findings of the bioinformatics analysis, thereby identifying hsa-miR-181a-5p as the most upregulated gene (Fig. 5B). We found that hsa-miR-181a slightly declined with age in normal BMD samples, but was significantly enhanced in osteoporotic bone tissues of old people (Fig. 5C). Using an ovariectomy-induced and an age-related osteoporosis murine model, we detected that the osteoclast-related gene expression (TRAP and DC-STAMP) and miR-181a expression were enhanced in both groups (Fig. 5D–F). Using normal and mutated luciferase reporter containing putative miR-181a target sites of DUSP6 revealed that a miR-181a mimic could suppress the normal DUSP6 luciferase activity, but had no effect on the activity of the mutated luciferase reported (Fig. 5G). Overall, these results demonstrated that miR-181a was enhanced under pathological conditions and that it inhibited the expression of DUSP6 in vitro.

### MiR-181a accelerates osteoclastogenesis in vitro

**Fig. 6:**
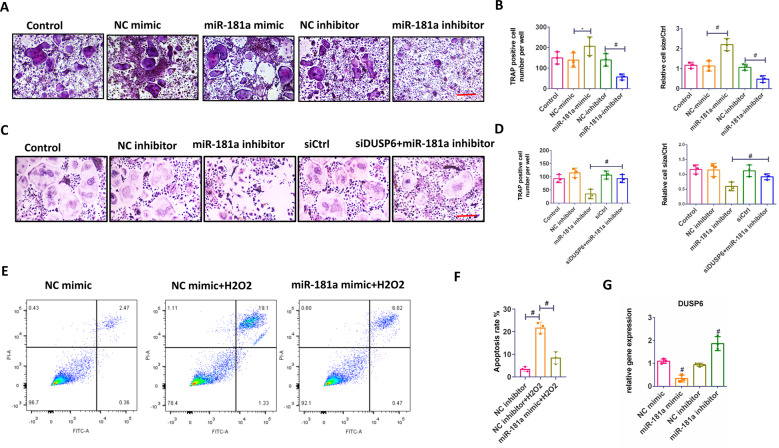
MiR-181a accelerated the osteoclastogenesis in vitro. **A** TRAP staining was carried out to explore the function of miR-181a during the osteoclastogenesis. **B** Quantitative analysis was established. **C** BMMs were transfected with NC inhibitor and miR-181a inhibitor. DUSP6 expression was silenced using RNAi. TRAP staining were carried out to evaluate the osteoclast differentiation in the indicated groups. Original scale bars: 200 μm. **D** Quantitative analysis were carried out to confirm the TRAP staining analysis. **E** The effect of miR-181a on osteoclast apoptosis was calculated using Annexin V-FITC/PI kit by a flow cytometer. **F** The expression of DUSP6 in the indicated groups was analyzed using real-time PCR in RNAKL-induced osteoclastogenesis. Data in all bar graphs are expressed as mean ± SD (*n* = 3). *P < 0.05, ^#^*P* < 0.01.

BMMs were treated with RANKL or RANKL with a negative control (NC) mimic, miR-181a mimic, NC inhibitor, and miR-181a inhibitor. The miR-181a mimic inhibited DUSP6 expression during RANKL-induced osteoclastogenesis and induced osteoclast differentiation, whereas the miR-181a inhibitor suppressed the process, as confirmed by TRAP staining and quantitative analysis (Fig. 6A, B). To determine the relationship between DUSP6 and miR-181a in RANKL-induced osteoclastogenesis, we silenced DUSP6 using RNAi and observed that suppressed DUSP6 attenuated the miR-181a inhibitor-mediated blockade of osteoclastogenesis (Fig. 6C, D). Moreover, miR-181a mimic greatly suppressed H_2_O_2_-induced osteoclast apoptosis (Fig. 6E, F). RT-PCR confirmed the regulation of miR-181a on the expression of DUSP6 (Fig. 6G).

### DUSP6 regulates osteoclastogenesis via the ERK2 signaling pathway

**Fig. 7:**
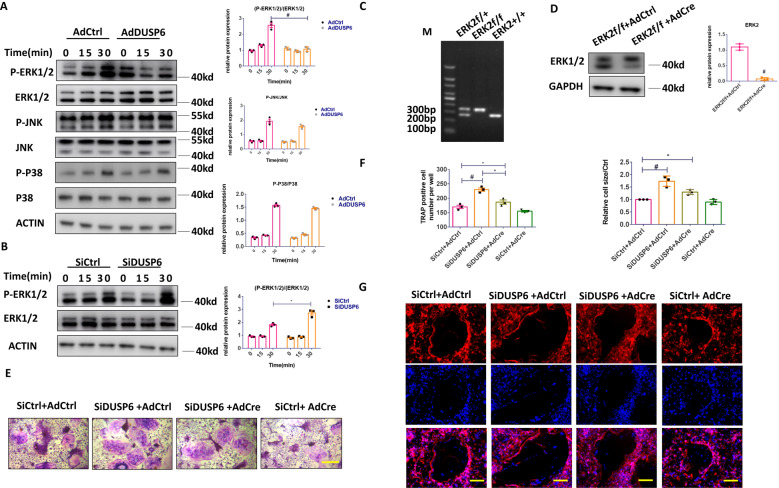
DUSP6 regulated the osteoclastogenesis via ERK2 signaling. RANKL-induced osteoclast differentiation was established. **A** BMMs were transfected with Ad-Control (AdCtrl) and Ad-DUSP6. And phosphorylation of ERK1/2, P38, and JNK signaling pathway was analyzed for the indicated time point. **B** BMMs were transfected with SiControl(SiCtrl) and SiDUSP6. And the phosphorylation of ERK1/2 was examined. **C** The genotyping analysis was carried out using PCR to confirm the ERK2 deletion according to the following standard: ERK2 wt: only 275 bp; ERK2f/+: 275 bp + 350 bp; ERK2f/f: only 350 bp. **D** BMMs were isolated from the ERK2f/f mice. And then they were transfected with Adcontrol(AdCtrl) and AdCre to delete the EKR2 expression. Western blot analysis was carried out to confirm this result. **E** BMMs isolated from the ERK2f/f mice were transfected to SiCtrl, SiDUSP6, AdCtrl, and AdCre in the indicated group. TRAP staining was carried out to explore the osteoclast differentiation. **F** Quantitative analysis was carried out. Original scale bars: 200 μm. **G** F-actin ring formation was carried out to explore the function of mature osteoclast. Original scale bars: 200 μm. Data in all bar graphs are expressed as mean ± SD (*n* = 3). *P < 0.05, ^#^*P* < 0.01.

Next, the mechanism involving DUSP6 in osteoclastogenesis was investigated. BMMs were transfected with adenovirus carrying DUSP6 to evaluate the ERK, JNK, and P38 signaling pathways. We observed that ERK signaling was markedly suppressed in BMMs overexpressing DUSP6, whereas DUSP6 inhibition enhanced the RANKL-induced ERK signaling (Fig. 7A, B). To confirm these results, BMMs were isolated from ERK2f/f mice and transfected with adenovirus-Cre to knockdown ERK2 (Fig. 7C, D). TRAP staining was performed to confirm the effect of ERK2 on DUSP6-mediated osteoclastogenesis, showing that DUSP6 RNAi enhanced osteoclast differentiation, whereas ERK2 deficiency could moderately attenuate this effect (Fig. 7E, F). F-actin ring formation confirmed that osteoclast maturation was suppressed by ERK2 deficiency (Fig. 7G). Overall, these data showed that DUSP6 moderately inhibited osteoclast differentiation via the ERK2 signaling pathway. Nonetheless, other signaling pathways might also be involved in this process.

### DUSP6 regulated osteoclastogenesis via the SMAD2 signaling pathway

**Fig. 8:**
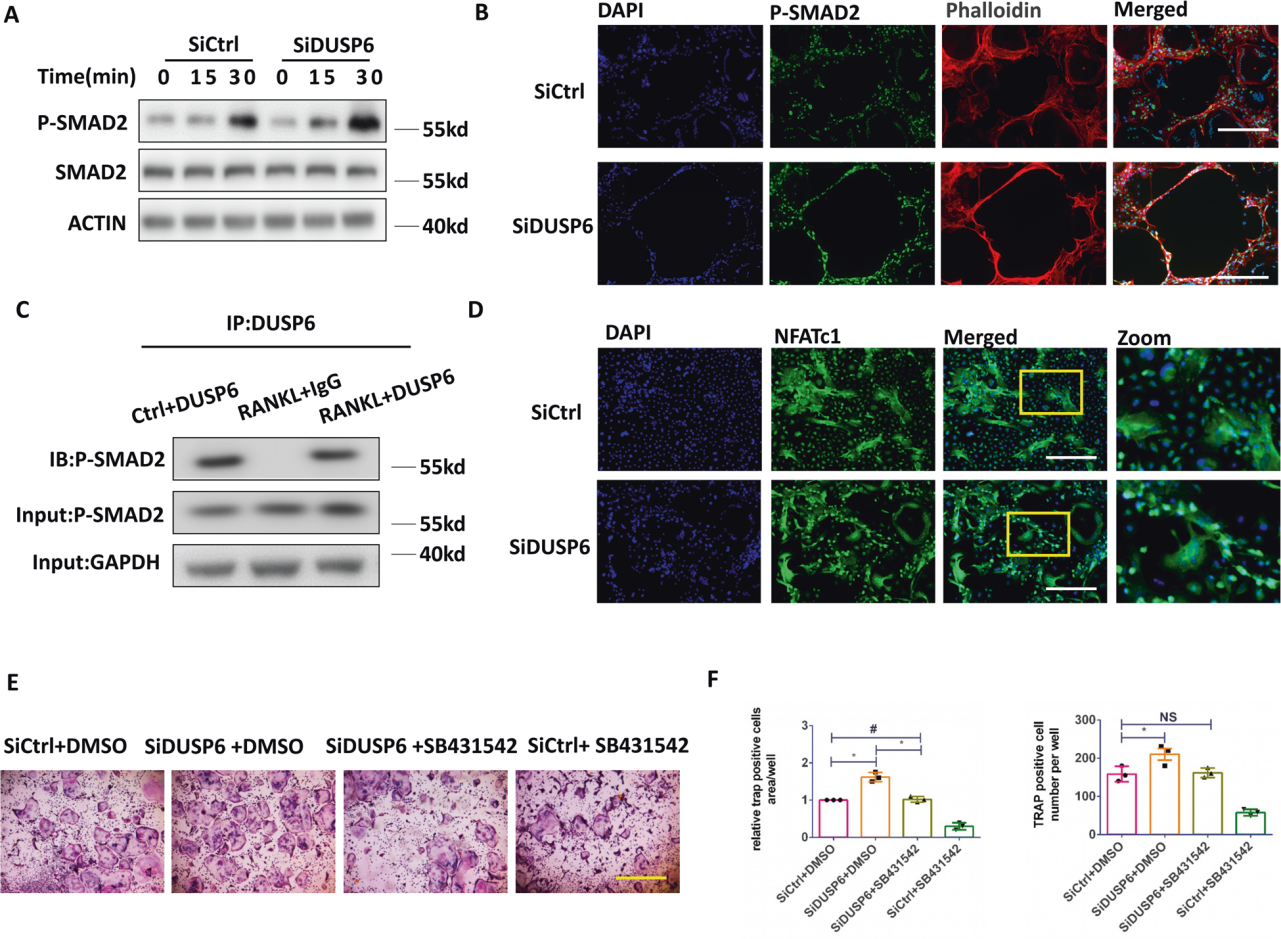
DUSP6 regulated the osteoclastogenesis via SMAD2 signaling. RANKL-induced osteoclast differentiation was established. **A** BMMs were transfected with SiControl (SiCtrl) and SiDUSP6. And the phosphorylation of SMAD2 was examined for the indicated time point. **B** Immunofluorescence assay was carried out to further explore the expression of P-SMAD2.F-actin ring formation was carried out to define the mature osteoclast. **C** Immunoprecipitation assay was carried out to clarify the connection between DUSP6 and P-SMAD2. **D** BMMs were transfected with SiCtrl and SiDUSP6. Immunofluorescence assay was carried out to detect the NFATC1 expression in the nucleus. **E** BMMs were transfected with SiControl (SiCtrl) and SiDUSP6. And they were then treated with the P-SMAD2 inhibitor SB31542 to further confirm the effect of P-SMAD2 in DUSP6-mediated osteoclastogenesis. TRAP staining (**E**) and quantitative analysis were carried out (**F**) to determine the osteoclast differentiation. Data in all bar graphs are expressed as mean ± SD (*n* = 3). Original scale bars: 200 μm.*P < 0.05, ^#^*P* < 0.01.

SMAD2 signaling in DUSP6-mediated osteoclastogenesis was explored by western blotting and immunofluorescence assays. DUSP6 RNAi was found to markedly enhance the RANKL-induced SMAD2 signaling (Fig. 8A, B). Immunoprecipitation revealed that phospho-SMAD2 signaling was involved in the nuclear transportation of NFATC1 and that suppression of DUSP6 by RNAi significantly induced the nuclear transportation of NFATC1 (Fig. 8C, D). Furthermore, the SMAD2 signaling inhibitor SB431542 was found to attenuate osteoclastogenesis mediated by DUSP6 inhibition, as confirmed by TRAP staining and quantitative analysis (Fig. 8E, F). Overall, we showed that DUSP6 inhibition induced osteoclast differentiation by enhancing SMAD2 phosphorylation and consequently promoting the nuclear transportation of NFATC1.

## Discussion

This is the first report on the role of DUSP6 in osteoclastogenesis via regulation of the ERK signaling pathway. We showed that DUSP6 directly regulates the SMAD2 signaling pathway in RNAKL-induced osteoclastogenesis. Evaluation of DUSP6 regulators identified miR-181a as a potential candidate. miR-181a was positively correlated with osteoporosis by regulating osteoclast differentiation and suppressing osteoclast apoptosis. Thus, DUSP6 might be a target for treating osteoclast-related diseases, such as osteoporosis and rheumatoid arthritis.

The DUSP subfamily inactivates target kinases, such as MAPK/ERK, SAPK/JNK, and p38, and consequently regulating cellular activity [8]. Specifically, DUSP6 inactivates ERK2 and regulates the related biological process [32]. Previous studies showed that the ERK/MAPK signaling pathway is involved in several physiological and pathological conditions, including osteoclast differentiation [33]. To date, no ERK kinase inhibitors were clinically approved. Noteworthy, several clinical-trials considered DUSP6 as a biomarker of the inhibition of the ERK/MAPK pathway (ClinicalTrials.gov identifier: NCT04305249, NCT01320085, and NCT02711345). The role of the DUSP subfamily in osteoclastogenesis remains unclear owing to the limited information available. Some studies reported that DUSP1, DUSP2, and DUSP5 are involved in osteoclast-related diseases, such as osteoporosis and rheumatoid arthritis, but the role of DUSP6 has not been studied. DUSP6 is proved to be involved in biological functions, such as M2 macrophage polarization [34], immunologicalreaction [35], aggressive malignant peripheral nerve sheath tumors (MPNSTs) growth, and T cell receptor sensitivity [17]. Therefore, further research is needed to clarify the role of DUSP6 in musculoskeletal-related diseases. Drugs designed for targeting DUSP6 activity have not been used for human application. Considering DUSP6 role in the regulation of ERK signaling, DUSP6 may represent a promising alternative for treating ERK signaling-related diseases.

In the present study, we used gene microarray analysis to demonstrate that DUSP6 is downregulated in human and mice osteoporotic bone tissues. We found that DUSP6 suppresses osteoclast differentiation and is negatively associated with bone density in vitro and in vivo. Overexpression of DUSP6 greatly inhibited the osteoclast differentiation in vitro, which was confirmed by trap staining (Supplementary Fig. S1C), bone resorption analysis (Supplementary Fig. S1D), and F-actin ring formation (Fig. S1E). In addition to the classic regulatory role of DUSP6 in the ERK/MAPK signaling pathway, a previous study indicated that DUSP6-mediated SUMOylation directly regulates Drp1 dephosphorylation and protects the cell from oxidative damage [15]. SMAD2-dependent NFATC1 regulation and osteoclast differentiation was observed [36]. In this study, we showed that DUSP6 suppresses SMAD2 phosphorylation, inhibiting the nuclear transportation of NFATC1 (Fig. 8 and Supplementary Fig. S1F–I). We also explored the RANKL-induced NF-kB signal pathway, which plays a vital role in osteoclastogenesis. We observed that DUSP6 did not affect the NF-κB signaling (Supplementary Fig. S1I). Furthermore, overexpression of DUSP6 attenuated bone loss in the ovariectomy-induced osteoporosis model (Supplementary Fig. S3). Thus, this study demonstrates for the first time that DUSP6 exerts osteoclastogenesis-inhibiting effects by directly regulating the ERK and SMAD2 signaling pathways.

Considering the differential mRNA expression between the osteoporosis and normal groups, we focused on the miRNAs to further explore the upstream DUSP6 regulation. Using bioinformatics, we identified miR-181a as a potential regulator of DUSP6 and confirmed that it was positively correlated with osteoporosis in human and mice samples. A previous study indicated that miR-181a declines with age in naive CD4(+) T cells [37]. Herein, we found that miR-181a slightly declined with age in normal bone tissues, but was markedly enhanced in the osteoporosis group. Therefore, abnormal miR-181a expression may be involved in pathological processes such as osteoporosis. Previous studies revealed that miR-181a regulates osteoclast apoptosis via FasL expression to maintain bone remodeling balance, and that locked nucleic acid miR-181a-5p antisense oligonucleotides attenuate osteoarthritis in knee joints. We demonstrated that miR-181a regulates DUSP6 expression, and consequently the ERK and SMAD2 signaling pathways, during osteoclast differentiation. Further research is still needed to clarify the specific role of miR-181a in osteoporosis in vivo using transgenic mice. Locked nucleic acid miR-181a-5p antisense oligonucleotides could also be analyzed for treating osteoporosis.

Osteoblasts play a critical role in the progression of osteoporosis [38]. Our previous study also focused on the role of osteoblasts in the process of osteoporosis [39]. Pre-osteoblastic cells MC3T3-E1 were transfected with Ad-DUSP6 and cultured for osteoclast differentiation. Alkaline phosphatase and Alizarin red staining indicated no significant changes between the Ad-DUSP6 and control groups. These results indicated that DUSP6 might not be involved in osteoblast differentiation (Supplementary Fig. S2D).

This study has some limitations. Additional experiments using DUSP6f/f-Lyzm-cre mice could provide valuable information on the role of DUSP6 in osteoporosis progression. In addition, the role of osteoblasts in DUSP6-mediated antiosteoporotic effect could be further investigated by performing calcein staining in vivo.

In summary, this study is the first to clarify the role of the miR-181a/DUSP6/ERK and SMAD2 axis in osteoclast differentiation and demonstrates that DUSP6 and miR-181a may be potential targets for the treatment of osteoclast-related diseases.

## Supplementary information

Supplementary Figure S1

Supplementary Figure S2

Supplementary Figure S3

Figure legend

## Data Availability

All data needed to evaluate the conclusions in the paper are present in the paper and/or the Supplementary Materials. Additional data related to this paper may be requested from the authors.
