# Supplementary figures and images for: DUSP6 expression is associated with osteoporosis through the regulation of osteoclast differentiation via ERK2/Smad2 signaling
Source: Cell Death Dis. 2021 Sep 2;12(9):825. doi: 10.1038/s41419-021-04110-y (PMC8413376; doi:10.1038/s41419-021-04110-y)

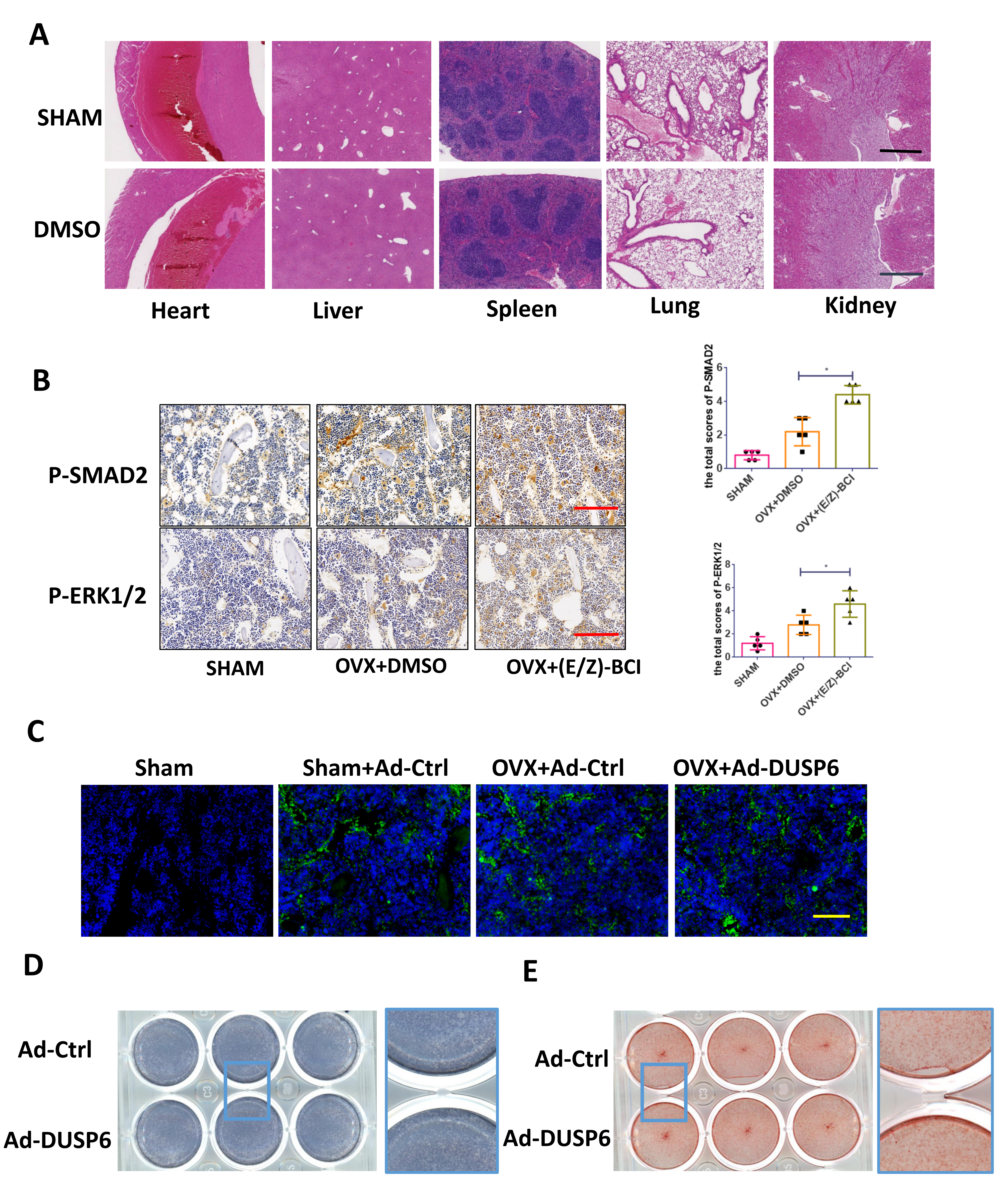

Supplement: Supplementary file 2 — Supplementary Figure S2 [file 41419_2021_4110_MOESM2_ESM.tif]

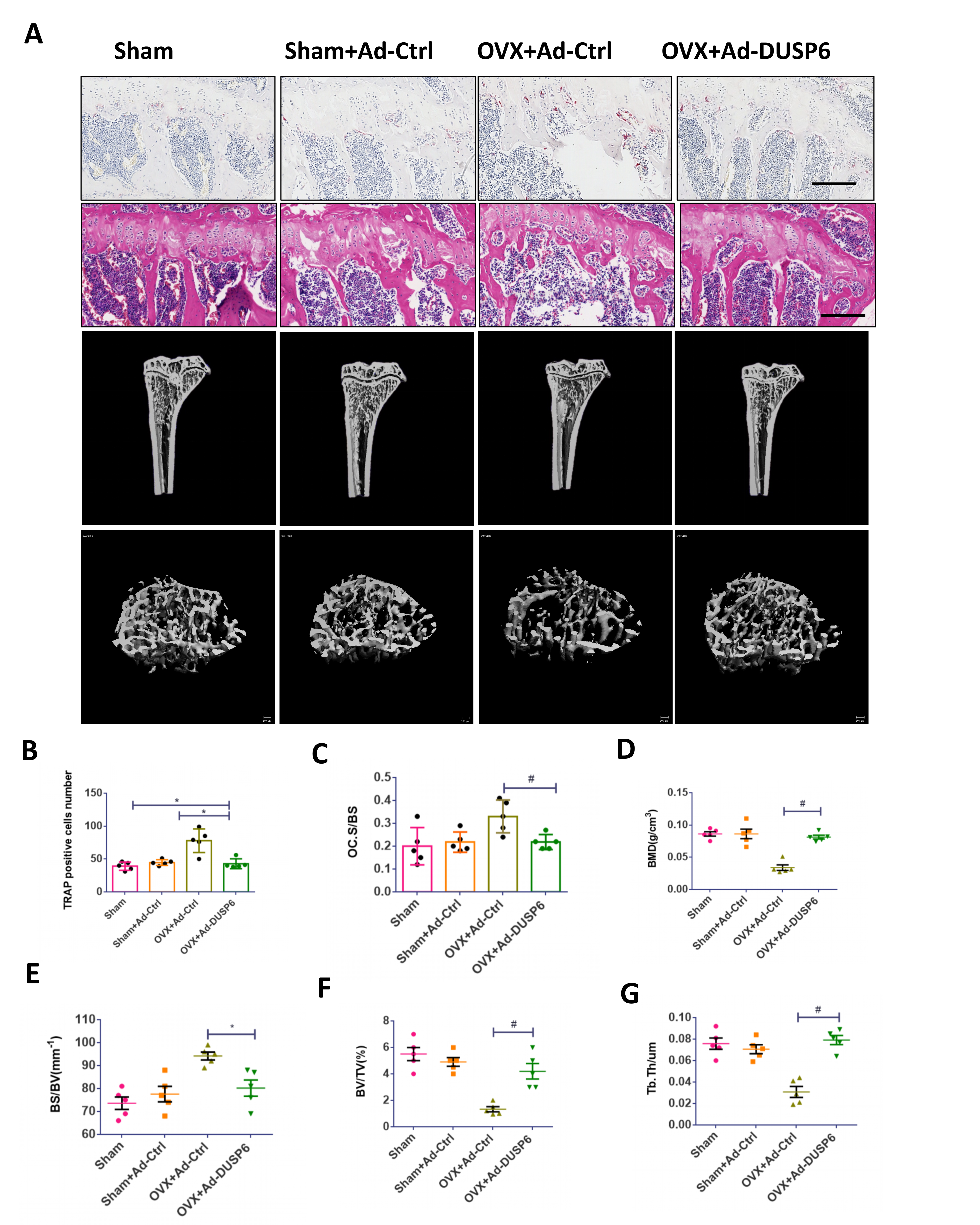

Supplement: Supplementary file 3 — Supplementary Figure S3 [file 41419_2021_4110_MOESM3_ESM.tif]
