# Supplementary material for: DUSP6 expression is associated with osteoporosis through the regulation of osteoclast differentiation via ERK2/Smad2 signaling
Source: Cell Death Dis. 2021 Sep 2;12(9):825. doi: 10.1038/s41419-021-04110-y (PMC8413376; doi:10.1038/s41419-021-04110-y)
Supplement: Supplementary file 4 — Figure legend [file 41419_2021_4110_MOESM4_ESM.pdf]

Figure legend for Supplementary Fig S 1

(A)Human normal and osteoporosis bone tissues were collected. The expression of GRAMD1B, ITGA6, and DUSP6 were analyzed using Real-time PCR. (B)BMMs were transfected with small interfering RNA (RNAi, #1, #2 and #3). The efficiency was confirmed by western blot analysis. (C) BMMs were transfected Ad-DUSP6.And the efficiency was confirmed by western blot analysis. BMMs were transfected Ad-DUSP6 and Ad-Ctrl. RANKL induced osteoclastogenesis was established. TRAP staining(D), bone resorption analysis(E) and F-actin ring formation(F) was analyzed. (G)The protein expression of Nfatc1 and C-Fos was detected in the indicated time point in RANKL induced osteoclastogeneiss using western blot. (H)The gene expression of Nfatc1 and C-Fos was detected in the indicated time point in RANKL induced osteoclastogeneiss using Real-time PCR. (I)Quantitatively analysis of western blot result. (J) BMMs were transfected Ad-DUSP6.RANKL was treated for the indicated time. And the expression of P-P65, P65, P-SMAD2, SMAD2 was analyzed using western blot. (K) BMMs were transfected with Ad-DUSP6 for the indicated time. The expression of CTSK was analyzed using western blot. Data in all bar graphs are expressed as mean ± SD. *P < 0.05, #P < 0.01.

Figure legend for Supplementary Fig S 2

(A)Normal C57 mice was intraperitoneal injection of 1% DMSO twice a week for 6 weeks. And HE staining was carried out to observe the lung, liver, spleen, heart and kidney in the two groups. (B) DUSP6 inhibitor (E/Z)-BCI was used in OVX induced OP model. The expression of P-ERK1/2 and P-SMAD2 was analyzed using immunohistochemistry analysis. (C) Ad-DUSP6 and Ad-Ctrl at a dose of 1×10^8 units was injected into the tail vein of mice once every 2 weeks for a total of 8 weeks. The tibia was collected to observe the transfection efficiency under fluorescence microscope. Transfected cells show green signal. Pre-osteoblastic cells MC3T3-E1 was transfected with Ad-DUSP6 and cultured for osteoclast differentiation. ALP staining(D) and Alizarin red staining(E) was used to detect the osteoblast differentiation. Data in all bar graphs are expressed as mean ± SD. *P < 0.05, #P < 0.01.

Figure legend for Supplementary Fig S 3

Experimental osteoporosis model was established. Mice were treated with Ad-DUSP6 or Ad-Control for analyzing the function of DUSP6 in vivo. (A) The tibia was collected from the indicated groups and TRAP staining was analyzed to detect the osteoclasts. HE staining was carried out to detect the bone structure in the indicated group. uCT was analyzed to confirm the bone loss. Quantitative analysis was used to determine the average TRAP-positive cell numbers from five different versions(B) and the TRAP-positive cell bone surface/bone surface(C). (D-G) The bone volume/tissue volume (BV/TV), trabecular thickness (Tb.Th), trabecular number (Tb.N), and trabecular separation (Tb.Sp) were measured to evaluate the microstructure. Original scale bars: 500 μm.Data in all bar graphs are expressed as mean ± SD(n = 5). *P < 0.05, #P < 0.01.
